# Provider Perspectives on the Influence of Family on Nursing Home Resident Transfers to the Emergency Department: Crises at the End of Life

**DOI:** 10.1155/2015/893062

**Published:** 2015-08-24

**Authors:** Caroline Stephens, Elizabeth Halifax, Nhat Bui, Sei J. Lee, Charlene Harrington, Janet Shim, Christine Ritchie

**Affiliations:** ^1^Department of Community Health Systems, School of Nursing, University of California, San Francisco, 2 Koret Way, N531E, UCSF Box 0608, San Francisco, CA 94143-0608, USA; ^2^Department of Geriatrics, Palliative & Extended Care, San Francisco VA Medical Center, Division of Geriatrics, School of Medicine, University of California, San Francisco, 4150 Clement Street, Building 1, Room 220F, San Francisco, CA 94121, USA; ^3^Department of Social & Behavioral Sciences, School of Nursing, University of California, San Francisco, 3333 California Street, Suite 455, UCSF Box 0612, San Francisco, CA 94118, USA; ^4^Division of Geriatrics, School of Medicine, University of California, San Francisco, 3333 California Street, Suite 380, San Francisco, CA 94143-1265, USA

## Abstract

*Background*. Nursing home (NH) residents often experience burdensome and unnecessary care transitions, especially towards the end of life. This paper explores provider perspectives on the role that families play in the decision to transfer NH residents to the emergency department (ED). *Methods*. Multiple stakeholder focus groups (*n* = 35 participants) were conducted with NH nurses, NH physicians, nurse practitioners, physician assistants, NH administrators, ED nurses, ED physicians, and a hospitalist. Stakeholders described experiences and challenges with NH resident transfers to the ED. Focus group interviews were recorded and transcribed verbatim. Transcripts and field notes were analyzed using a Grounded Theory approach. *Findings*. Providers perceive that families often play a significant role in ED transfer decisions as they frequently react to a resident change of condition as a crisis. This sense of crisis is driven by 4 main influences: insecurities with NH care; families being unprepared for end of life; absent/inadequate advance care planning; and lack of communication and agreement within families regarding goals of care. *Conclusions*. Suboptimal communication and lack of access to appropriate and timely palliative care support and expertise in the NH setting may contribute to frequent ED transfers.

## 1. Introduction

Transfers of frail nursing home (NH) residents to and from the emergency department (ED) are common and costly and expose vulnerable residents to the well-documented risks associated with care transitions [1–7]. Despite increasing legislation to monitor and enforce NH standards and quality of care over the past decade, the number of ED visits by NH residents has increased 12.8%, from 1.9 million to 2.1 million visits, and there has been no significant change in rates of potentially preventable ED visits [8]. Many of these burdensome care transitions occur in the last 6 months of life [9, 10] or are for symptoms or conditions that can be safely and effectively treated in the NH setting [11–14]. Unfortunately, many NHs not only lack adequate staffing and access to timely on-site medical expertise [6, 15, 16] but also have little to no access to palliative care support and services [17–19].

Experts believe that a key element to preventing avoidable hospitalizations and ED visits is early engagement of families in discussions about advanced care planning (ACP) and reducing nonbeneficial care at the end of life (EOL) [6]. Some have postulated that NH resident families are often poorly informed about their loved one's prognosis as well as treatment options available in the NH setting, therefore opting for a transfer to the hospital as the perceived most reasonable alternative [20]. Several empirical studies suggest that families have a significant influence on hospital transfer decisions [21–25]. However, little is qualitatively known about the nature of this family influence, particularly from the perspective of diverse health care providers across care settings.

The focus of this paper is to describe providers' perspectives on the role that families play in the decision to transfer NH residents to the ED. Findings reported here are part of a larger qualitative study that conducted eight 2-hour focus group interviews to explore diverse stakeholder perspectives on (1) the experiences and challenges they faced with NH resident transfers to the ED and (2) the potential role of technology to help to address those concerns. During the course of data collection for this larger study, the NH and hospital providers consistently spoke about the strong influence family members had on transfer decisions. This emerging concept was included in subsequent iterations of the interview guide and was found to be one of the main themes derived from the data.

## 2. Methods

### 2.1. Study Design

This descriptive study employed a qualitative approach using focus group interviews. Focus groups were deemed to be the most efficient method to collect data from this diverse group of individuals that included busy health professionals [26, 27]. In addition, focus groups were considered a useful method for triggering additional stories, examples, and thoughts from participants through the process of listening to one another and being reminded of issues they had not previously considered or remembered [28].

### 2.2. Sample and Setting

Participants were recruited via multiple methods: in-person meetings and/or emails with NH administrators, medical directors, and/or directors of nursing, as well as hospital/ED administrative leadership; posting of recruitment posters in local facilities and hospitals; emails to local and state-wide NH listservs; and snow ball sampling. Eligible participants were all English-speaking individuals involved in the care of a NH resident transferred to the hospital in the prior 3–6 months.

Table 1 reveals the demographics and characteristics of the 35 provider focus group participants. The sample included 16 NH nurses (including bedside nurses, charge nurses, directors of nursing, minimum data set coordinators, and a director of staff development); 11 primary care providers (including 6 NH physicians, 4 nurse practitioners (NP), and 1 physician assistant); 2 ED physicians; 2 ED nurses; 3 NH administrators; and 1 hospitalist. Forty percent of provider stakeholders were over the age of 46, 60% were female, and 46% were white. Approximately half of all providers had more than 15 years of clinical experience.

Some of the focus groups included mixed stakeholders (e.g., physician/nurse practitioner), while others were homogenous in membership (e.g., all nurses). No study participant participated in more than one focus group. The majority of groups took place in a private room at a public library in the San Francisco Bay Area.

### 2.3. Procedures

After informed consent was obtained and prior to the start of the focus group, participants were provided with an iPad to complete a brief web-based survey. This survey asked questions regarding participant sociodemographic characteristics, as well as background, experiences, and opinions about NH resident ED transfers.

Using a semistructured interview guide, the principal investigator (CS) then facilitated the focus group, and the study coordinator (EH) took field notes and made observations. During the first half of the focus groups (the findings of which are the focus of this paper), each participant was asked to describe a recent experience or experiences they had had with a NH resident who became ill and potentially required a transfer to the ED. As described earlier, NH and hospital providers consistently spoke about the strong influence family members had on transfer decisions and this emerging concept guided refinements to the semistructured interview guide. Probes used to elicit more information regarding perceptions of family involvement are shown in the following list:

- Please describe what the communication with the family was like during that time.
- How was the family engaged in the decision about the possible care transition?
- Did family engagement occur before or after the decision to transfer had been made?
- Please describe how the resident's goals of care were considered in the care transition?

### 2.4. Analysis

Data saturation was reached when no new concepts or perspectives were introduced during the focus groups [29, 30]. All interviews were recorded and professionally transcribed verbatim and then checked for accuracy. Using a Grounded Theory approach [29, 30], analysis began with coding immediately after data collection began, including initial coding of text line by line. No a priori codes were used. Categories were created from related codes and themes were developed based on patterns that described the phenomenon of interest. Interview guides for subsequent focus groups were modified to incorporate some of the issues emerging in early analysis. To ensure trustworthiness of this study, several strategies were employed. These included methodological transparency; collection of rich and sufficient data from a wide range of informants; and use of reflexivity by the research team to examine their preconceptions and biases. Approval for this study was obtained from the institutional review board at the authors' university.

## 3. Findings: Influence of Family

Families were commonly perceived as the major reason NH residents experienced potentially unnecessary transfers to the hospital. Providers reported that families often reacted to resident changes in condition as crises and that this precipitated their efforts to push for hospital transfers. Providers often described family influence as a tension between resident/family autonomy and their own clinical judgment when it came to making transfer decisions. For example, one NH nurse practitioner commented:

*“I think the biggest reason [for NH-ED transfer] is the family piece - because you can know whatever you know, and want to do whatever you want to do, but if mom or brother or sister or DPOA (durable power of attorney), or whatever, doesn't agree with that, it's very difficult to change their mind.” [NP]*

NH health care providers further related that they often made transfer decisions against their best clinical judgment because of this family influence and for fear of potential repercussions (e.g., complaints, lawsuits, and poor outcomes). An example of this tension is described here by a NH physician:

*“They wanted to take him to the hospital, clearly, but I didn't want that…family has a lot of influence. The family upset at the bedside and pushing for a transfer to the hospital - not doing that is going to lead to more problems in the long run, and the patient is certainly going to end up in the hospital. One way or another, they're going to end up in the hospital with an angry family member rather than a family member who feels like you've addressed their concerns.” [physician] *

The tensions around transfer between NH staff and families were also recognized by hospital providers who were often on the receiving end of inappropriate NH transfers:

*“So it wasn't necessarily [that] they [NH care team] didn't do the right thing; they tried, but I think they had issues with the patients.” [physician] *

### 3.1. Reacting to a Change of Condition as a Crisis

Underpinning this complex phenomenon of family influence on hospital transfer decisions was the perception that families reacted to a resident's change of condition as a crisis. Often this sense of crisis or panic led families to change care decisions resulting in transfers, usually at the EOL.

*“The residents that are long-term residents, some of those, I find, it's a family problem, because they will say “we don't want any heroics. Mom says I never wanted a tube, I never wanted this”, and so they fill out the POLST [Physician Orders for Life-Sustaining Treatments]. And then when we call and say—…‘their condition has changed a lot', they get panicked… there have been times where the family just panics and goes, ‘oh, my God, I didn't mean to do that, let me send her in [to the emergency department (ED)]'.” [nurse] *

*“I had a lady who just—her heart stopped—and that was it, and there wasn't anything that came before that, and she was totally no code, no anything, but when we called the family to tell the daughter, she said, “no, no, no, start CPR, get that going”. So we did, which we hadn't done, because she was a no CPR, so we had nurses in there doing CPR on her.” [nurse]*

As described in more detail below, this sense of family crisis or panic was driven by 4 main influences, largely stemming from unmet palliative care needs. These 4 dynamic and interdependent influences included insecurities with NH care; being unprepared for EOL; absent or inadequate advance care planning; and lack of communication and agreement within families regarding goals of care.

#### 3.1.1. Insecurities with NH Care

Providers perceived that families often felt insecure about the care provided in the NH setting and how that frequently seemed to influence family preference for ED transfer. Such insecurities were largely driven by families questioning the quality of NH licensed nurse care and perceiving NH medical capacity and responses as inadequate (e.g., lack of physician support/communication, inability to get timely test results, and failure to provide timely analgesics). For example, NH and hospital providers described that families often insisted on transfer from NH to ED because they felt their loved one's needs were not being met in the NH setting:

*“… patients are brought in [to the ED] because the family is saying, “well, they're not taking care of your pain”, or “I'm not getting whatever I need”, or conditions are…below their expectations I guess. So that seems to be another challenge.” [physician] *

Providers further described that this feeling of insecurity and drive for hospital transfer was often exacerbated when families came to visit and perceived that their loved ones were not being sufficiently cared for. For example, one nurse described that transfers were often high on days when families frequently came to visit:

*“Sunday evenings are big for skilled nursing facility transfers, because families come to visit and they don't like what they're seeing.” [nurse]*

One physician described how these insecurities with NH care would sometimes lead families to change the resident's code status:

*“I'm not quite sure how that happens [hospital DNR patients reverting to full code on return to NH]. I think part of it is that in the hospital, they'll [patient/family] accept a DNR status, because they feel like they're in an acute [care] setting, but that the family members change their mind, because they're afraid that if they're DNR in a nursing home, that not enough will be done. So they make themselves full code while they're in the nursing home, and then with the understanding they'll probably switch back to DNR when they get to the acute.” [physician]*

#### 3.1.2. Being Unprepared for End of Life

Many providers perceived that families often seemed emotionally unprepared for their loved one's decline in health and ultimate death. Participants portrayed families who often did not understand or recognize the needs of those at the EOL. Descriptions emerged of family members whose actions were commonly driven by denial about their resident's health status. One nurse described how a resident's sister had an unrealistic understanding of her sister's prognosis:

*“She had pulmonary issues…numerous co-morbidities, so she was constantly going in [to the ED]…She had a sister that took care of her and she was not a do-not-resuscitate. Her sister wanted everything done for her…she wasn't brain dead, but the sister was insisting that she was able to communicate with her, and she'd talk to her. But there was nothing [she was profoundly demented]…it was kind of sad. We always thought that if she [the resident] could wake up and say, “you know, I don't want this”, that she probably would, but her sister was her primary caregiver and wanted everything possible done for her. So we went with her sister's wishes.” [nurse] *

In describing two terminally ill residents, this nurse tells how the residents did not receive hospice care as recommended but died in the ED, because family were not ready to transition to a symptom-oriented, palliative approach to care:

*“…they [family] were not ready at that time to initiate hospice, so one day he really exacerbated, he went to the hospital, CHF exacerbation, and he expired at the hospital…We have had two of these instances recently: one was metastatic lung cancer, and the other was CHF. And they were both in one month. They [the families] were just not ready at that time for hospice, even though we saw it. We did the consultation, the notes were there from hospice that they're ready physically, however, mentally and psychologically, they were not ready to give up yet.” [nurse]*

Another nurse described a resident at EOL who had frequent transfers to and from the hospital because the son could not “let her go.” This nurse, however, did not blame the resident's son for being unprepared for EOL but rather faulted the lack of good communication and discussion between the physician and the family.

*“…we have one long, long, long, long-term son who sends his mother to the emergency room probably once a month at least, and won't let her go. And the emergency room doctors cannot do anything with him; neither can all the other doctors. So they're used to seeing him, they don't do anything more; it's just a trip back and forth. And some of these things could be, I think, [pause] handled better if the physician who is responsible—the attending physician—had more of a relationship with the family.” [nurse] *

#### 3.1.3. Absent or Inadequate Advanced Care Planning

Driving this feeling of panic that influenced families was absent or inadequate advance care planning discussions and decisions. Without medical orders reflecting treatment decisions, providers articulated that the default was to provide all available medical treatment, including ED transfer. The process for making ACP decisions at the NH was described as being centered on the completion of the POLST. However, the process of completing the POLST was described as being largely driven by and completed by admissions coordinators, social services, and/or nursing staff and then the form was left for the physician to sign. Physicians were perceived to be minimally involved in discussing and completing the POLSTs with residents and families: what one nurse described as simply providing a “*red stamp signature only.*” Several NH nurses described the frustration of taking on responsibility for initiating the POLST forms whilst chasing physicians to help manage residents' and families' expectations and sign off to complete them. Following are a few examples of the process for completing the POLST from the nursing perspective:

*“…as an IDT [Interdisciplinary team], we work together. And when the doctor is there, we grab the doctor and everything, get him to sign the form right away.” [nurse]*

*“…we will tell the doctor, you know, doctor, you've got to explain to the family about her condition that this is, you know, what's the goal.” [nurse]*

While lack of physician involvement was implicated as a cause of the absence or inadequate advance care planning, focus group participants also perceived families were frequently reluctant to make what one physician referred to as “hard decisions.”

*“…but a lot of times the families, you're lucky to get them to fill out square one [of the POLST], because they don't want to make that decision. It's like, ‘well, call me when that happens'.” [nurse]*

* “[You] present it [POLST] right to them, okay, now we have a box A, B, and C, can you check one of these and tell us what would be your best answer, or your best directive, and they won't do it.” [nurse]*

Advance care planning was also considered inadequate in situations where POLSTs used were described as “confusing,” “incomplete,” or “contradictory.” The conflict between DNR and full treatment orders caused real frustration for this ED physician:

*“…[advanced care planning is] a continuous and ongoing issue, not just in terms of patients who don't have POLSTs, but POLSTs that are filled out incorrectly, or POLSTs that are confusing. If you check somebody that is DNR, but then you say full treatment on the second column, that makes no sense.” [physician]*

#### 3.1.4. Lack of Communication and Agreement within Families regarding Goals of Care

Lack of communication and agreement within families regarding goals of care was another major influence on the decision to transfer a resident to the ED. One nurse stated that family members are often “*not on the same page.*” At times when residents experienced a change of condition, this lack of communication led to disagreements about what treatments/transfers were in the resident's best interests and a disregard for existing advanced care orders. As described in this next quote, this was often exacerbated when the resident had children locally involved and others engaged at a distance:

*“…there's a brother [out of state] who wants [ED transfer], and a sister who doesn't…we deal with a tremendous number of family members who are at great odds with each other on end-of-life situations, on transfer to the hospital.” [nurse]*

In a separate example, a nurse described a resident who had become unresponsive. She checked the POLST that stated she had a Do Not Resuscitate (DNR) order. She called the doctor and daughter, but she was unable to reach them.

*“…she had a son and a daughter, and they were both responsible party. So the son told me, ‘oh, it's DNR, keep her there, keep her comfortable'. Okay, so I'm going to follow that…and then I got a call from the doctor who said if they want her out [to the ED], go ahead and send her out…The daughter comes running in…‘oh, send her now'. I was like your brother said – ‘don't listen to him' (said the daughter) – so it's like communication between the two and it's like something they should have worked…out before, you know, when that actual moment happened. And so yeah, she ended up, a couple days later, passing away [in the hospital], but the communication is horrible.” [nurse].*

Nurses frequently described personal stress and frustration when the resident's family members gave contradictory directions regarding goals of care in times of crisis.

*“So as far as…[advanced care planning] is concerned, I think that…nurses are…always in, you know, between a rock and a hard place, especially with the families that have the conflict.” [nurse]*

*“I can think now, probably 5 people with families, that one person has been appointed, the ‘primary', or like ‘executor person' that will be making the decisions. And then you'll have the other – maybe two other family members coming in at different times during the week, or during the month and it's like ‘no one told me about my dad', or ‘no one told me this about my mom', and it's just a hard place to be in…it's like well, your sister is the primary decision-maker, and you're not listed here, so that would be up to her to notify you about this. Because then they start in with ‘you never called me, you never told me', and it's just a hard place to be as a nurse.” [nurse]. *

Many of these family communication challenges and conflicts regarding goals of care were rooted in the complex interplay of the above 4 main influences. The dynamic and interdependent nature of these influences ultimately led to residents and their families having unmet palliative care needs. Such unmet needs included lack of preparation for EOL, inadequate facilitation of goals of care conversations, and lack of advance care planning.

## 4. Discussion

Providers perceived that families play a significant role in ED transfer decisions as they frequently react to a resident change of condition as a crisis. This sense of crisis was driven by 4 dynamic and interdependent factors: insecurities with NH care; being unprepared for EOL; absent or inadequate advance care planning; and lack of communication and agreement within families regarding goals of care. These findings are congruent with other research citing family insistence [6, 31–34], as well as concerns about advanced care directives [6, 35], as common nonmedical factors associated with hospital transfer decisions. This is the first study to highlight how such family influence on ED transfer decisions is often driven by a sense of crisis and panic, largely stemming from unmet palliative care needs. The lack of access to appropriate and timely palliative care support and expertise in the NH setting may be a contributing factor to frequent and often burdensome ED transfers at the EOL. Many unnecessary transfers could be reduced with improved goals of care discussion, increased sense of security with symptom management, and basic care needs, as well as greater anticipatory guidance regarding what to expect with disease progression, prognosis, and needs at the EOL [36, 37].

Such provision of palliative care, or specialized medical care for people with serious illnesses, has been strongly advocated by experts as a way to enhance the care for vulnerable NH residents with progressive, life-limiting illness and late-life disability [37–40]. Evidence suggests this high risk population has many unmet needs for symptom amelioration, provider communication, emotional support, and being treated with respect, especially near the end of life [9, 10, 39]. As a person-centered model of care that integrates individualized psychosocial and physical care to enhance quality of life for residents and families [40, 41], palliative care may help proactively address the factors identified in this study that precipitated family feelings of crisis. Evidence suggests that NH palliative care is associated with improved care quality and satisfaction, enhanced symptom management, and fewer ED visits [42–45]. Unfortunately, this vulnerable population has historically had little access to formal palliative expertise outside of hospice services [17–19].

Findings further suggest a need to reframe the negatively perceived role of families in hospital transfer decisions. Conceptualizing families as one of the major drivers of inappropriate transfers run the risk of making them adversaries rather than partners in care. Numerous studies underscore the importance and value of eliciting resident perspectives and actively engaging them and their families in shared decision-making [33, 36, 37, 46, 47]. Such “patient-centered care” is a central tenet of the Institute of Medicine (IOM) report, Affordable Care Act and the national NH culture change movement [48–50], and is posited to improve health outcomes and reduce or eliminate any disparities associated with access to needed care and quality [50–52].

Consistent with other studies [32, 53–56], many of the perceived challenges that participants perceived with families often stemmed from limited physician involvement in proactively engaging families in goals of care discussions. A growing body of evidence suggests, however, that there are larger systems issues that inhibit such provider efforts, as well as provision of quality EOL care in general in the NH setting [57]. Moreover, systematic processes to elicit goals of care in NHs are lacking [47, 57, 58]. A recent review article concluded that NH residents wish to be involved in EOL related decisions; however family and staff do not always recognize resident preferences or ability to consent to preferences [59]. Missed opportunities for communication about EOL preferences among NH residents, family, and staff are common [47]. Towsley and colleagues [47] argue that these “missed conversations” occur when no one inquires about EOL preferences; residents, families, or staff assume wishes are known; and resident information is not conveyed due to the lack of a formalized process to converse about or share resident wishes.

Providers in this study highlighted significant challenges with goals of care discussions in general and as documented with the POLST in particular. Some POLST studies indicate that open conversations between NH residents, family members, and medical providers can increase comfort at the EOL, reduce hospitalizations, and increase the likelihood that residents' and families' preferences are solicited, documented, and honored [60–63]. Unfortunately, as this and other studies [64–66] have found, POLST completion often becomes more of an administrative function (e.g., check the boxes and have the doctor sign) versus a platform for providers to engage in an actual discussion regarding goals of care. It is ultimately not about the form but rather the process and it appears that that process may be getting lost in the NH setting.

As Sudore and Fried [67] suggest, ACP needs to occur “upstream,” years earlier when individuals have decision-making capacity and are able to voice their wishes to their family. They recommend reframing the purpose of ACP away from having patients and families make premature treatment decisions based on incomplete information; rather ACP should entail “preparing patients and their surrogates for the types of decisions and conflicts they may encounter when they do have to engage in in-the-moment decision-making” [67]. Such ACP discussions are not a one-time occurrence but rather an ongoing process that needs to be frequently revisited and documented as individual preferences and circumstances may change over time.

ACP discussions in the NH setting are increasingly focused on completing the POLST (or related document in other states) [68, 69]. While it is important to have a completed POLST form on the chart and one that accompanies a resident during care transitions, it is only useful and appropriate if it accurately reflects current treatment preferences. More qualitative documentation is needed regarding the nature and content of the ACP discussions as they evolve over time. Greater efforts should be made to move beyond just “checking a box” and properly train all staff on how to engage in these important conversations and appropriately complete POLST forms [70–72]. Staff should think creatively about how to change “missed conversations” into opportunities that involve families, residents, and staff and elicit or account for resident preferences [47]. Since NH residents are in close proximity to other residents and often witness EOL situations first hand, the experience of others could be used as a way to initiate EOL conversations [47, 73]. Involving nurse practitioners in care planning conferences may also represent an excellent opportunity to revisit goals of care with residents and their families in the context of broader interprofessional treatment planning [74].

*Limitations*. This study has some important limitations worth noting. The goal of the larger study was to better understand the range of challenges our sample of stakeholders experienced in the care of this vulnerable population and how emerging health technologies might help address those concerns. It is therefore not clear how subject preference to participate or not participate in a technology-oriented focus group may have influenced responses received. In addition, the study sample was relatively small and only included perspectives of providers across 3 counties in northern California. Providers in other parts of the state and country might have different experiences and perspectives. Nevertheless, rich qualitative data were obtained from a wide range of providers who worked in diverse settings, including for-profit/not-for-profit and large chain/small chain facilities located in both urban and suburban settings primarily serving ethnically and racially diverse communities.

## 5. Conclusions

A growing body of evidence suggests that NH residents experience many detrimental and costly care transitions, particularly at the EOL [75–77]. These findings underscore the strong need for greater upstream anticipatory guidance and preparation for “in-the-moment” decision-making to reduce family conflict, crisis, and panic when there is a change in condition. Moreover, study findings further suggest that improved access to timely and appropriate palliative care support and expertise in the NH setting may reduce many of these burdensome and unnecessary transitions at the EOL.

With greater national efforts focused on providing person-centered care, improving care transitions, and reducing health care costs for frail older adults in NHs, policymakers and payers should recognize the value of high quality palliative care in the NH setting. It is also critical to establish appropriate and adequate reimbursement mechanisms for these vital but often time-consuming and sensitive resident and family conversations regarding goals of care [74]. Future research is needed to better understand the specific palliative care needs of nursing home residents and their families. Moreover, as hospitals and NHs work more closely together to reduce inappropriate care transfers and improve value-based care, innovative care strategies that improve access to much needed palliative care services and supports for NH residents need to be evaluated.

## Figures and Tables

**Table 1:** Demographics and characteristics of provider focus group participants (*N* = 35).

Age (years)	
18–35	6 (17%)
36–40	8 (23%)
41–45	7 (20%)
46–50	3 (9%)
Over 50	11 (31%)
Gender	
Male	14 (40%)
Female	21 (60%)
Race	
American Indian/Alaska Native	1 (3%)
Asian	11 (31%)
Native Hawaiian/other Pacific Islanders	1 (3%)
Black or African American	1 (3%)
White	16 (46%)
More than one race	1 (3%)
Unknown/not reported	4 (11%)
Healthcare role	
NH nurse	16 (46%)
NH physician	6 (17%)
Nurse practitioner/physician assistant	5 (14%)
Emergency department nurse	2 (6%)
Emergency department physician	2 (6%)
NH administrator	3 (9%)
Hospitalist	1 (3%)
Years of experience in clinical practice/NHs	
1–5	8 (23%)
6–10	7 (20%)
10–15	3 (9%)
More than 15	17 (49%)
